# Cost‐effectiveness of preventive aspirin use and intensive downstaging polypectomy in patients with familial adenomatous polyposis: A microsimulation modeling study

**DOI:** 10.1002/cam4.6488

**Published:** 2023-08-30

**Authors:** Eiko Saito, Michihiro Mutoh, Hideki Ishikawa, Kenichi Kamo, Keisuke Fukui, Megumi Hori, Yuri Ito, Yichi Chen, Byron Sigel, Masau Sekiguchi, Osamu Hemmi, Kota Katanoda

**Affiliations:** ^1^ Institute for Global Health Policy Research Bureau of International Health Cooperation National Center for Global Health and Medicine Tokyo Japan; ^2^ Institute for Cancer Control National Cancer Center Tokyo Japan; ^3^ Department of Molecular‐Targeting Prevention, Graduate School of Medical Science Kyoto Prefectural University of Medicine Kyoto Japan; ^4^ Department of Molecular‐Targeting Cancer Prevention, Graduate School of Medical Science Kyoto Prefectural University of Medicine Osaka Japan; ^5^ Center for Medical Education Sapporo Medical University Sapporo Japan; ^6^ Graduate School of Advanced Science and Engineering Hiroshima University Hiroshima Japan; ^7^ School of Nursing University of Shizuoka Shizuoka Japan; ^8^ Department of Medical Statistics, Research & Development Center Osaka Medical and Pharmaceutical University Takatsuki City Osaka Japan; ^9^ Division of Health Medical Intelligence, Human Genome Center, The Institute of Medical Science The University of Tokyo Tokyo Japan; ^10^ Cancer Screening Center/Endoscopy Division National Cancer Center Hospital Tokyo Japan; ^11^ Division of Screening Technology National Cancer Center Institute for Cancer Control Tokyo Japan; ^12^ Department of Health Promotion National Institute of Public Health Saitama Japan

**Keywords:** aspirin, chemoprevention, colorectal cancer, cost‐effectiveness analysis, familial adenomatous polyposis, microsimulation

## Abstract

**Objective:**

Although there is increasing evidence to suggest the cost‐effectiveness of aspirin use to prevent colorectal cancer (CRC) in the general population, no study has assessed cost‐effectiveness in patients with familial adenomatous polyposis (FAP), who are at high risk of developing CRC. We examined the cost‐effectiveness of preventive use of low‐dose aspirin in FAP patients who had undergone polypectomy in comparison with current treatment practice.

**Design:**

We developed a microsimulation model that simulates a hypothetical cohort of the Japanese population with FAP for 40 years. Three scenarios were created based on three intervention strategies for comparison with no intervention, namely intensive downstaging polypectomy (IDP) of colorectal polyps at least 5.0 mm in diameter, IDP combined with low‐dose aspirin, and total proctocolectomy with ileal pouch‐anal anastomosis (IPAA). Cost‐effective strategies were identified using a willingness‐to‐pay threshold of USD 50,000 per QALY gained.

**Results:**

Compared with no intervention, all strategies resulted in extended QALYs (21.01–21.43 QALYs per individual) and showed considerably reduced colorectal cancer mortality (23.35–53.62 CRC deaths per 1000 individuals). Based on the willingness‐to‐pay threshold, IDP with low‐dose aspirin was more cost‐effective than the other strategies, with an incremental cost‐effectiveness ratio of $57 compared with no preventive intervention. These findings were confirmed in both one‐way sensitivity analyses and probabilistic sensitivity analyses.

**Conclusion:**

This study suggests that the strategy of low‐dose aspirin with IDP may be cost‐effective compared with IDP‐only or IPAA under the national fee schedule of Japan.

## Graphical abstract

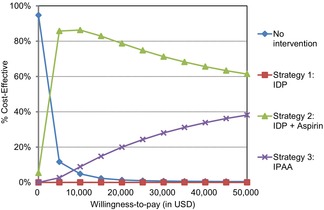

## 1 INTRODUCTION

Familial adenomatous polyposis (FAP) is a genetic disease caused by germline mutations in the *adenomatous polyposis coli* (*APC*) gene and is characterized by the development of 100 or more adenomas (polyps) in the colorectum.^1^ The incidence of FAP in the general population is estimated to be one to two in 20,000 individuals in Western countries, and one in 17,400 individuals in Japan.^2^ Less than 1% of colorectal cancer patients are estimated to have FAP.^3^ It has been reported that polyps found in the colorectum are likely to progress to adenocarcinoma in almost 50% of FAP patients by the age of 40 years and that if not treated, most patients with FAP develop colorectal cancer by the time they reach 60 years old.^1^ While it is not possible to predict which among the 100 or more polyps will become malignant, in the case of colorectal cancer, polyp size is a significant risk factor associated with carcinogenesis. It is considered that polyps which increase in size with age are more likely to undergo malignant transformation.^4^

The standard treatment for FAP is prophylactic total colectomy with resection of the entire large intestine.^5^ Total colectomy often carries a poor prognosis and adversely impacts the quality of life due to frequent diarrhea and incontinence,^6^ infecundity in women,^7^ and the development of desmoid tumors.^8^ In Japan, total proctocolectomy combined with ileal pouch‐anal anastomosis (IPAA) is currently considered the standard surgical procedure and has been increasingly implemented.^9^ Endoscopic management of FAP via repeated colonoscopies to remove polyps greater than 5 mm in size, called Intensive Downstaging Polypectomy (IDP), has been reported to reduce a polyp burden,^10^, ^11^ yet IDP has not been fully recommended as a prophylactic modality to date; nor has the use of non‐surgical approaches to treat FAP, such as chemopreventive agents.

Recent clinical trials have consistently reported the effect of nonsteroidal anti‐inflammatory drugs (NSAIDs), particularly aspirin, in preventing sporadic colorectal adenoma in Western^12^ and Asian populations.^13^ Further, administration of low‐dose aspirin has been shown to reduce recurrent adenomas of more than 5.0 mm in diameter in FAP patients who had previously undergone endoscopic removal of colorectal polyps of 5.0 mm or more.^14^ This suggests that aspirin inhibits the growth of colorectal polyps, which is a risk factor for the development of colorectal cancer. Given that aspirin is widely available at low cost, the prophylactic use of aspirin in preventing recurrent adenomas in FAP patients may be worth investigating in terms of cost‐effectiveness. Although accumulating evidence has suggested the cost‐effectiveness of aspirin use with colonoscopic surveillance in the chemoprevention of colorectal cancer in a general population,^15^, ^16^ no study has yet investigated the cost‐effectiveness of low‐dose aspirin in FAP patients.

Microsimulation models are a convenient method that can estimate the long‐term impact of different intervention strategies that cannot be compared in the real world, taking account of the heterogeneities in risks by participant background, mortality over time, and population dynamics.^17^ Here, we examined the potential cost‐effectiveness of preventive use of low‐dose aspirin in FAP patients who had undergone polypectomy in comparison with current treatment practice using microsimulation modeling.

## 2 METHODS

### 2.1 Model description

**FIGURE 1:**
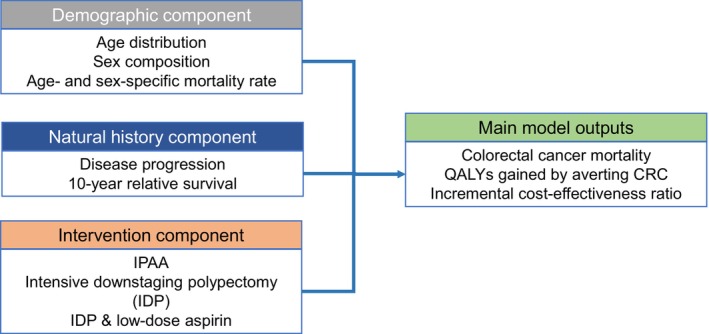
Structure of the microsimulation model.

**FIGURE 2:**
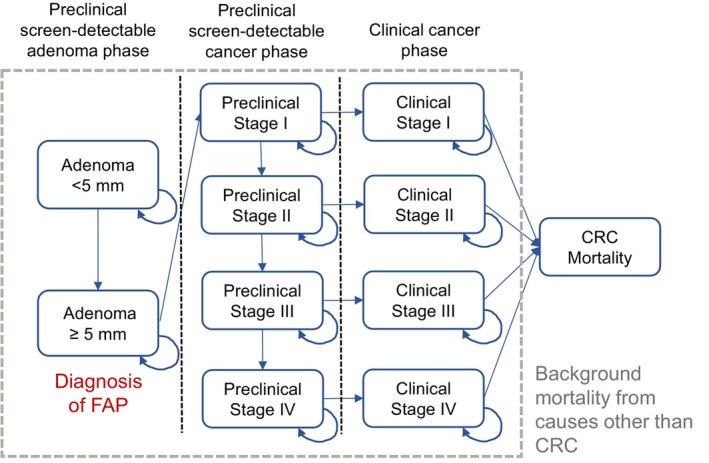
Natural history of the microsimulation model. Note: Arrows indicate transition from one state to another (or remain in the same state).

Our model consisted of three components—a demographic component, a natural history component, and an intervention component (Figure 1). To construct the demographic component, we obtained information from nationally representative datasets on demographics,^18^, ^19^ cancer incidence, and mortality.^20^ We used this data to develop a microsimulation model of colorectal cancer in FAP patients and create a virtual population with sex‐ and age‐specific mortality from causes other than colorectal cancer. The natural history of disease progression was constructed following the adenoma‐carcinoma sequence (Figure 2).^4^ FAP patients carry a large number of colorectal polyps at the time of diagnosis,^1^ and these polyps may develop into colorectal cancer at some point in life depending on the model input parameters (Table 1).

**TABLE 1:** Key input parameters included in the analysis.

Input parameter	Base‐case analyses	One‐way sensitivity analyses (range)	Probabilistic sensitivity analyses (distribution)	Source
Natural history—transition probabilities (per year)				
Prevalence of FAP in the general population	0.00006	Fixed	Fixed	[2]
From high‐risk polyp to preclinical stage I CRC in FAP patients^a^	0.015982 (<30 years); 0.113758 (30+ years)	Fixed	Fixed	[24]
From preclinical stage I to preclinical stage II CRC^a^	0.24022	Fixed	Fixed	[24]
From preclinical stage II to preclinical stage III CRC^a^	0.22290	Fixed	Fixed	[24]
From preclinical stage III to preclinical stage IV CRC^a^	0.27487	Fixed	Fixed	[24]
From preclinical stage I to clinical stage I CRC^a^	0.07202	Fixed	Fixed	[25]
From preclinical stage II to clinical stage II CRC^a^	0.11011	Fixed	Fixed	[25]
From preclinical stage III to clinical stage III CRC^a^	0.18989	Fixed	Fixed	[25]
From preclinical stage IV to clinical stage IV CRC^a^	0.87577	Fixed	Fixed	[25]
Probability of survival in CRC patients^b^	0.0729–1.000	Fixed	Fixed	[23]
Colonoscopy surveillance—test characteristics				
Sensitivity for 5–9 mm low‐risk polyp	0.86500	Fixed	Fixed	[45]
Sensitivity for high‐risk polyp (10+ mm)	0.97600	Fixed	Fixed	[45]
Specificity for colorectal polyps and CRC	1.00000	Fixed	Fixed	[45]
Probability of perforation after colonoscopy without polypectomy	0.00010	Fixed	Fixed	[46]
Effectiveness				
Probability of developing polyps (5+ mm) after IDP	0.554	Fixed	Fixed	[35]
Probability of developing polyps (5+ mm) after IPAA (once in a lifetime)	0.036	Fixed	Fixed	[47]
Relative risk of 1 or more polyps (5+ mm) incidence with aspirin and IDP	0.37	0.16–0.86	Beta	[14]
Adverse effects				
Probability of nausea, vomiting, stomach pain, and diarrhea grade 1–2 due to aspirin (for initial 3 days)	4.00%	Fixed	Fixed	[14]
Probability of harms associated with IDP—perforation	0.40%	Fixed	Fixed	[11, 35]
Probability of harms associated with IDP—intestinal bleeding	2.30%	Fixed	Fixed	[11, 35]
Utility loss (QALYs)				
Per colonoscopy^c^	−0.003	Fixed	Fixed	[36]
Per complication of colonoscopy with polypectomy^d^	−0.019	Fixed	Fixed	[36]
Per LY with CRC care				
Stage I—initial care	−0.120	Fixed	Fixed	[36]
Stage I—continuing care	−0.050	Fixed	Fixed	[36]
Stage II—initial care	−0.180	Fixed	Fixed	[36]
Stage II—continuing care	−0.050	Fixed	Fixed	[36]
Stage III—initial care	−0.240	Fixed	Fixed	[36]
Stage III—continuing care	−0.240	Fixed	Fixed	[36]
Stage IV—initial care	−0.700	Fixed	Fixed	[36]
Stage IV—continuing care	−0.700	Fixed	Fixed	[36]
Per ileal pouch‐anal anastomosis (IPAA)	−0.110	Fixed	Fixed	[37]
Per serious gastrointestinal bleed/ulcer/aphtha grade 1–2 due to aspirin^e^	−0.004	Fixed	Fixed	[48]
*Discounting* (*Costs and QALYs*)	3.00%	0–4%	Fixed	[28]
Cost (US$)^f^				
Surveillance and surgery				
Screening colonoscopy	145.2	116–174	Gamma	[49]
Colorectal surgery (IPAA)	17,540	14,032‐21,048	Gamma	HICD
IDP for FAP patients	1222.5	978–1467	Gamma	HICD
Chemoprevention				
Aspirin (100 mg/day for 1 year × Markov cycle)	19.5	16–23	Gamma	HICD
CRC treatment				
Stage I—1st year	12,367	Fixed	Fixed	[49]
Stage I—2nd to 5th year	333	Fixed	Fixed	[49]
Stage II—1st year	13,109	Fixed	Fixed	[49]
Stage II—2nd to 5th year	333	Fixed	Fixed	[49]
Stage III—1st year	21,929	Fixed	Fixed	[49]
Stage III—2nd to 5th year	421	Fixed	Fixed	[49]
Stage IV—1st year	25,178	Fixed	Fixed	[49]
Stage IV—2nd to 5th year	23,846	Fixed	Fixed	[49]
Treatment of adverse effects				
Perforation due to colonoscopy with endoscopic resection	12,376.9	9901‐14,852	Gamma	HICD
Intestinal bleeding due to endoscopic resection	2169.7	1736‐2604	Gamma	HICD

Abbreviation: HICD: Health Insurance Claim Database.

^a^ Probabilities are calibrated from the original input values as shown in Table S1.

^b^ 10‐year net survival probabilities vary by stage, sex and year after diagnosis.

^c^ Equal to 2 days per colonoscopy at a utility of 0.

^d^ Complications associated with hospitalization with 30 days of colonoscopy were assumed to be equal to 14 days at a utility of 0.5.

^e^ Gastrointestinal event for 3 days at a utility weight of −0.54.

^f^ All costs were reported in 2020 US dollars at an average exchange rate of 106.725 JPY/dollar and adjusted to the national fee schedule as of 2020.

Preclinical colorectal cancer is an undetected cancer that may either become symptomatic, be detected by screening, or progress through stages I to IV.^21^, ^22^ Using long‐term survival data from population‐based cancer registries, the survival time of individuals after cancer diagnosis was simulated by sex, clinical stage at diagnosis, and years after diagnosis.^23^ Background mortality from causes other than colorectal cancer was also modeled by age, sex, and calendar year.

Because the model estimation needs to match the real‐world incidence and mortality among FAP patients in Japan, we calibrated the natural history model against the absolute risk of colorectal cancer by age group provided by Iwama et al. (2004)^1^ and evaluated the model fit by goodness‐of‐fit testing, and the best‐fit parameter values were used in the final model (Supplemental Table S1).^24^, ^25^ The model was then validated against the lifetime probability of colorectal cancer death in FAP patients derived from Iwama et al. (1993).^26^ The model was developed using TreeAge Pro 2022 (TreeAge Software Inc). Data were analyzed using Stata 16.1 (Stata Corp.). The details of the model are described in Supplemental Document 1.^1^, ^4^, ^18^, ^19^, ^20^, ^23^, ^27^

### 2.2 Intervention strategies

We evaluated three intervention strategies as scenarios for comparison against the base case scenario, namely (1) the base case, in which no preventive intervention is performed; (2) intensive downstaging polypectomy (IDP)—i.e. endoscopic removal of all colorectal polyps with a diameter of 5.0 mm or greater; (3) IDP combined with low‐dose aspirin to prevent recurrent adenoma^14^; and (4) total proctocolectomy with ileal pouch‐anal anastomosis (IPAA).^5^ The baseline natural history was modeled to predict the probability of developing colorectal cancer in the absence of any preventive interventions. Any preventive intervention can alter the natural history of colorectal cancer by reducing the probability of recurrent adenoma and removing existing adenoma of 5.0 mm or greater in diameter; or by detecting preclinical cancer, depending on the sensitivity and specificity of the surveillance. All three intervention strategies were administered to FAP patients aged 16 years and above. Individuals aged 16 years and above with IDP or IPAA were assumed to undergo annual surveillance colonoscopy after intervention. Perfect adherence to surveillance was assumed in all strategies. We assumed that low‐dose aspirin use would be halted if gastrointestinal bleeding were observed, and that all colorectal cancer patients with FAP should undergo the standard treatment for sporadic colorectal cancer, which includes surgery and chemotherapy, or IPAA or IRA with regional lymph node dissection, in accordance with the Japanese Society for Cancer of the Colon and Rectum (JSCCR) guidelines for the Clinical Practice of Hereditary Colorectal Cancer (2020).^5^

### 2.3 Cost‐effectiveness analysis

The cost‐effectiveness analysis was performed from a healthcare payer perspective, in which the model simulated a cohort of 100,000 individuals who were alive in 1963 and followed for 40 years (cycles) unless they died before the end of the simulation cycle. Effectiveness (quality‐adjusted life‐years [QALYs] gained) and costs were simulated for each intervention separately. Incremental cost‐effectiveness ratios (ICER) were calculated by dividing the incremental cost by the QALYs gained. A strategy would be dominated if it were more costly but yielded fewer QALYs than the other strategies; or if it incurred greater cost but the same QALYs as the other strategies. Costs and QALYs were discounted at an annual rate of 3%.^28^ A willingness‐to‐pay (WTP) threshold of $50,000 US dollars per QALY gained was applied.^29^ Table 1 presents the main probabilities, relative risks, and costs used in the model. All costs were reported in 2020 US dollars at the average exchange rate of 106.725 JPY/USD, and adjusted to the national fee schedule as of 2020. Supplemental Table S2 presents a Consolidated Health Economic Evaluation Reporting Standards (CHEERS) 2022 checklist.^30^

### 2.4 Sensitivity analysis

To assess the robustness of our modeled estimates to changes in model input parameters, we first performed one‐way deterministic sensitivity analyses by changing the cost inputs of aspirin, IDP, cost of treatment for intestinal bleeding, cost of perforation treatment, cost of annual surveillance colonoscopy, and cost of IPAA by changing the respective costs by ±20%. We also assessed the effects of uncertainty surrounding the relative risk of polyp recurrence after IDP and low‐dose aspirin. A discount rate of 3% per annum to both costs and effects was applied throughout all analyses, but we also explored a discount rate of 0% to 4% for both costs and effectiveness in our deterministic sensitivity analysis.^28^ Further, we evaluated the robustness of our results by changing the adherence rates of daily low‐dose aspirin intake from 80% to 95% based on the results from a systematic review of clinical trials.^31^

A probabilistic sensitivity analysis was also performed using a Monte Carlo simulation to investigate the effect of parameter uncertainty on the results. The model was run 100,000 times, each taking a random draw from input values with the predefined uncertainty distributions listed in Table 1 (Probabilistic sensitivity analyses [distribution]).

## 3 RESULTS

### 3.1 Calibration and validation results of the simulation model

**FIGURE 3:**
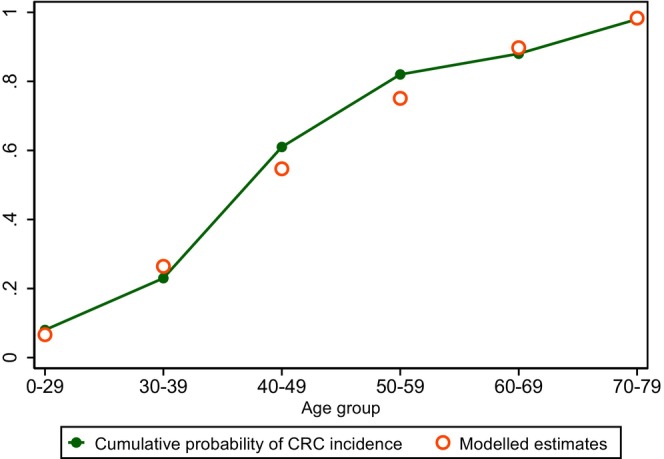
Model calibration results comparing the cumulative probability of CRC incidence (observed) versus modeled estimates. The observed probability of CRC incidence is shown with a green line, while the modeled estimates are shown with red hollow dots.

Our microsimulation model accurately estimated the cumulative risk of colorectal cancer incidence throughout all age groups below 80 years. However, the middle‐aged population (40–59 years old) showed estimates that were slightly more conservative than the CRC incidence reported by the Polyposis Committee of the Japanese Society for Cancer of the Colon and Rectum^1^ (Figure 3). Our external validation using the lifetime probability of CRC death also showed a high level of concordance (modeled estimate; 0.31958) compared with the reported probability (Iwama et al. 0.31905).^26^

### 3.2 Incremental cost‐effectiveness ratios

The model estimated that having no preventive intervention resulted in 325.3 colorectal cancer deaths per 1000 individuals and 19.19 QALYs per individual. Compared with no intervention, all strategies resulted in an increase in QALYs (21.01–21.43 QALYs per individual) and a considerable reduction in colorectal cancer mortality (23.35–53.62 CRC deaths per 1000 individuals). The average cost per person over 40 years—including the cost of preventive measures, annual surveillance colonoscopy, and colorectal cancer treatment—was highest for the strategy with IDP‐only ($19,314 per individual). In contrast, costs were lower for the IPAA strategy ($19,096 per individual) and for the combined IDP and low‐dose aspirin strategy ($11,100 per individual). Details of these results are shown in Table 2.

**TABLE 2:** Estimated incremental cost‐effectiveness of colorectal cancer prevention strategies in FAP patients.

	CRC deaths (per 1000)	QALYs (per person)^a^	Average cost (per person)^a^	ICER per QALY gained
No intervention	325.26	19.19	10,972	—
Strategy 1 (IDP)	53.62	21.01	19,314	Dominated^b^
Strategy 2 (IDP & Aspirin)	30.42	21.37	11,100	59^c^
Strategy 3 (IPAA)	23.35	21.43	19,096	122,796^d^

Abbreviations: CRC, colorectal cancer; ICER, Incremental Cost‐Effectiveness Ratio; IPAA, Ileal Pouch‐Anal Anastomosis; QALY, Quality‐Adjusted Life‐Year.

^a^ Discounted at an annual rate of 3%.

^b^ Dominated with greater cost and fewer QALYs than Strategy 2.

^c^ Compared against no intervention.

^d^ Compared against Strategy 2.

Incremental cost‐effectiveness ratios (ICERs) showed that the IDP‐only strategy was substantially less cost‐effective than the combined IDP and low‐dose aspirin strategy. Specifically, the IDP‐only strategy showed greater cost and a lower QALY gain than IDP with low‐dose aspirin. Compared to no intervention, the IDP and low‐dose aspirin strategy showed a minimal increase in average cost with increased QALYs, which resulted in an ICER of $59. The IPAA strategy had increased QALYs with a higher mean cost than the combined IDP and aspirin strategy, which resulted in an ICER of $122,796 for the IPAA strategy. Based on a willingness‐to‐pay (WTP) threshold of $50,000 per QALY gained, the IDP and low‐dose aspirin strategy was cost‐effective.

### 3.3 Sensitivity analysis

**FIGURE 4:**
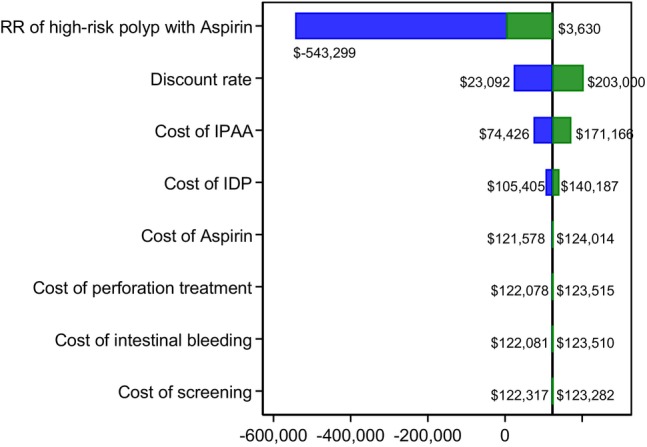
Tornado diagram showing ICER of IPAA over the strategy with IDP and low‐dose aspirin from one‐way sensitivity analyses. ICER from the base case analysis is shown in the straight black line. (Note: one‐way sensitivity analysis by applying a lower range of the relative risk of having 1 or more polyps (5+ mm) recurrence by having aspirin with IDP resulted in negative ICERs because the IPAA strategy had fewer QALYs gained and higher costs than the strategy with IDP and low‐dose aspirin).

In our one‐way sensitivity analyses, the combined IDP and low‐dose aspirin strategy remained cost‐effective after changing the key parameters. The ICERs of the IPAA over the IDP and aspirin strategy varied from $‐543,299 to $203,000 per QALY gained (Figure 4). The parameters that affected the ICERs of the IDP and aspirin strategy ranked from greatest to least effect, were relative risk of high‐risk polyp recurrence with aspirin; discount rate; cost of IPAA; cost of IDP; cost of aspirin; cost of perforation treatment; cost of treating intestinal bleeding; and cost of screening. Our sensitivity analysis of changing the adherence rates of aspirin intake also showed consistent results given the WTP threshold of $50,000 (Supplemental Table S3).

**FIGURE 5:**
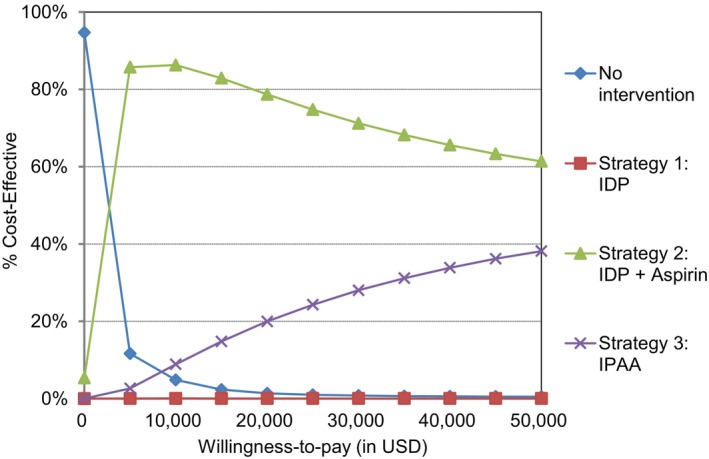
Cost‐effectiveness acceptability curves show the probability that a strategy is cost‐effective given a range of threshold values.

**FIGURE 6:**
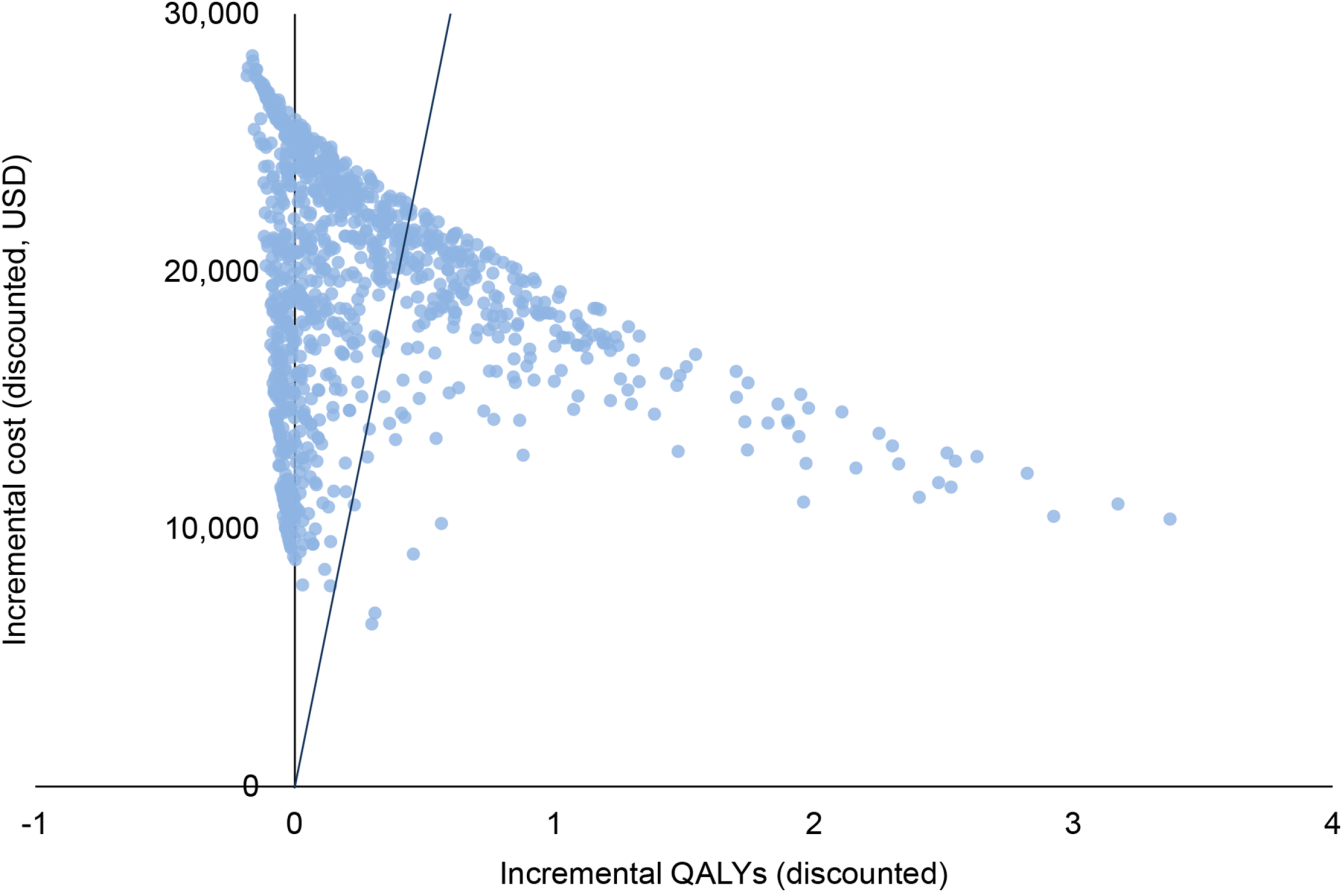
Incremental cost‐effectiveness scatterplot estimated from the probabilistic sensitivity comparing the IPAA strategy against the strategy with IDP and aspirin. Each dot represents the discounted incremental cost and incremental QALYs of one bootstrap sample. The black line denotes the willingness‐to‐pay threshold at USD50,000 per QALY gained.

In our probabilistic sensitivity analysis, the combined IDP and aspirin strategy was cost‐effective over the IPAA strategy in 74.8% of simulations based on a WTP threshold of $50,000; and in 61.4% of simulations with a WTP threshold of $100,000 (Figure 5). Although 67.8% of the simulations yielded additional QALY gains for the IPAA strategy over the IDP and aspirin strategy (Figure 6), 43.5% of the simulations resulted in an ICER higher than $50,000, and 32.2% of the simulations showed that the IPAA strategy was absolutely dominated by the IDP and aspirin strategy.

## 4 DISCUSSION

This study provides the first evidence for the cost‐effectiveness of prophylactic aspirin use in FAP patients who had previously undergone IDP. Our microsimulation analyses found that preventive use of low‐dose aspirin combined with IDP was more cost‐effective than IPAA in FAP patients given the willingness‐to‐pay threshold of $50,000 per QALY gained. The use of aspirin with IDP was also likely to be cost‐effective compared with the IDP‐only strategy.

Our results are consistent with data from clinical trials, which showed the benefits of administering aspirin in suppressing recurrent adenomas in FAP patients.^14^, ^32^, ^33^ The mechanism by which a regular dose of aspirin inhibits tumorigenesis can be explained by the results of immunohistochemical staining that epidermal growth factor receptor (EGFR) and cyclooxygenase‐2 (COX‐2) are concomitantly overexpressed in epithelial cells of the colorectal polyps of FAP patients, but that both overexpressions are attenuated by a regular dose of aspirin.^34^ Regarding the development of advanced cancer, a previous randomized control trial (RCT) that investigated the efficacy of aspirin use in FAP after IDP reported no occurrence of advanced cancer in either the control or intervention group due to frequent surveillance and endoscopic resection of polyps during the 8‐month follow‐up,^14^ yet a single‐arm intervention for FAP patients with IDP reported the occurrence of submucosal invasive CRC in 5 years (0.9%).^11^

The strategy with IDP and aspirin combined was found to produce greater cost saving per person on average than having no intervention, including with regard to daily use of aspirin, treatment of adverse effects, annual surveillance, and treatment of colorectal cancer. This is because the national fee schedule of Japan sets the cost of IDP in FAP patients at only around 1222.5 US dollars per surgery, regardless of the number of polyps removed, and also because aspirin is relatively inexpensive (19.5 US dollars per year). The high average cost per person for the IDP‐only strategy is attributable to the cost of repeated IDP, and the increased incidence of colorectal cancer. As for the IPAA, the high cost of IPAA surgery has increased the average cost per person; and with an annual discount rate of 3% per year, the costs of intervention that takes place immediately—such as IPAA—outweighs the cost of cancer treatment that may occur in the distant future.

Our results also showed a large reduction in colorectal cancer deaths in all three preventive strategies. Nevertheless, the gains in QALYs were not as pronounced as that in the magnitude of deaths averted (19.19 QALYs per person in the no‐intervention group vs. 21.07 to 21.43 QALYs per person in the intervention groups). This is partly due to discounting at an annual rate of 3%, which decreases the value of QALYs in the distant future. Furthermore, the expected QALY gains in the intervention groups were likely suppressed by utility loss due to adverse events, such as perforation and intestinal bleeding after IDP^35^; nausea, vomiting, stomach pain, and diarrhea due to aspirin^13^; utility loss per individual colonoscopy^36^; and IPAA.^37^ Further, IDP, which requires advanced endoscopic techniques, has only recently been implemented.^10^, ^11^, ^38^ Given the possibility of future reports on adverse events, continuous monitoring and improvement of study accuracy are important considerations.

Preventive use of low‐dose aspirin was initially recommended by the U.S. Preventive Services Task Force (USPSTF) in 2016 for the primary prevention of colorectal cancer, with specific targeting to those aged 50 to 59 years with high cardiovascular risk, no elevated bleeding risk, more than 10 years of life expectancy, and ability to use low‐dose aspirin every day for 10 years.^39^ The updated USPSTF guideline in 2022 retracted this recommendation because of limited evidence of effectiveness and considerable variation in results.^40^ However, these USPSTF recommendations pertain to the general population with a given health condition and predefined age group and have not been specifically targeted to high‐risk populations, such as patients with FAP or Hereditary Non‐Polyposis Colorectal Cancer (HNPCC). In Japan, some prophylactic treatments of cancer ‐ such as prophylactic mastectomy in patients with hereditary breast and ovarian cancer ‐ have been listed in the national fee schedule, which regulates all medical costs for reimbursement and is revised on an item‐by‐item basis biennially.^41^ Although IDP has been listed in Japan's national fee schedule since 2022, the regulatory approval for the use of aspirin in FAP patients has not been granted. Given that cost‐effectiveness plays a role in determining regulatory approval and the fee schedule, our findings, which highlight the cost‐effectiveness of IDP combined with aspirin therapy, could potentially inform the approval procedure for aspirin use.

The strength of this study lies in its use of a well‐validated microsimulation model that incorporated a natural history model of colorectal cancer in FAP patients, and the impact of preventive strategies on disease progression. This incorporation was achieved by synthesizing comprehensive evidence from nationally representative datasets and meta‐analyses. The model has been calibrated to replicate the probability of colorectal cancer incidence in FAP patients. A further strength is our use of multiple sensitivity analyses to show that the cost‐effectiveness of the combined IDP with low‐dose aspirin strategy over other strategies is robust given the uncertain range of the input parameters.

Some limitations also warrant mention. First, although we used postoperative utility scores and addressed a wider range of short‐ and long‐term adverse effects,^37^ this study did not consider the increased risk of desmoid tumor following surgery in FAP patients due to a paucity of data. It has been reported that 5% of male patients and 11% of female patients with FAP develop desmoid tumors postoperatively,^1^ and that the risk of desmoid tumors is similar between IPAA and ileorectal anastomosis (IRA).^42^ Second, although duodenal adenomas of Spigelman Stage IV or higher in FAP patients pose a risk of developing duodenal cancer^43^, ^44^ which may require surgery, we were unable to include a scenario for duodenal adenomas in our analysis due to a lack of data. For the same reason, we were unable to assess the effect of low‐dose aspirin after IRA or IPAA. Third, we based the effects of low‐dose aspirin on a clinical trial that administered aspirin for 8 months in Japanese patients, and the effects and safety of aspirin in the longer term have not been considered, nor have those of other factors such as ethnicity or degree of obesity.^14^ Current smokers in the general population were reported to be at increased risk of colorectal tumor development with regular low‐dose aspirin.^13^ However, we were unable to stratify our analyses by smoking status due to a paucity of data on FAP patients. However, we used the relative risk of recurrent tumor development by aspirin intake after adjustment of smoking status, and hence the possibility that we overestimated the benefit of aspirin is minimal. Finally, we modeled only the direct medical costs of colorectal cancer treatment and prevention and did not include societal costs associated with productivity loss. For the non‐intervention group, colorectal cancer treatment would lead to a considerable loss of work days, whereas for the intervention groups, surveillance colonoscopy, IDP, and IPAA would lead to a loss of productivity. Future studies warrant further investigation into the effects of prevention strategies on the indirect costs of prevention of colorectal cancer in FAP patients.

In conclusion, the present study showed that a strategy of low‐dose aspirin combined with IDP and annual surveillance colonoscopy is more cost‐effective than IDP‐only or IPAA under the framework of the national fee schedule of Japan. These findings highlight the need for further research on the prophylactic use of aspirin in people at high risk of colorectal cancer.

## AUTHOR CONTRIBUTIONS

**Eiko Saito:** Conceptualization (equal); formal analysis (equal); funding acquisition (equal); investigation (equal); methodology (equal); validation (equal); visualization (equal); writing – original draft (equal); writing – review and editing (equal). **Michihiro Mutoh:** Conceptualization (equal); data curation (equal); investigation (equal); supervision (equal); writing – review and editing (equal). **Hideki Ishikawa:** Conceptualization (equal); investigation (equal); methodology (equal); supervision (equal); writing – review and editing (equal). **Kenichi Kamo:** Investigation (equal); supervision (equal); writing – review and editing (equal). **Keisuke Fukui:** Investigation (equal); supervision (equal); writing – review and editing (equal). **Megumi Hori:** Data curation (equal); methodology (equal); writing – review and editing (equal). **Yuri Ito:** Investigation (equal); writing – review and editing (equal). **Yichi Chen:** Data curation (equal); writing – review and editing (equal). **Byron Sigel:** Data curation (equal); writing – review and editing (equal). **Masau Sekiguchi:** Investigation (equal); writing – review and editing (equal). **Osamu Hemmi:** Investigation (equal); writing – review and editing (equal). **Kota Katanoda:** Conceptualization (equal); investigation (equal); supervision (equal); writing – review and editing (equal).

## CONFLICT OF INTEREST STATEMENT

KKatanoda received a JMWH Bayer Grant (1 million JPY) from September 1, 2017 to August 31, 2019 via the Japan Society for Menopause and Women's Health. The authors declare no other conflicts of interest.

## ETHICS STATEMENT

Not applicable since this study used published data.

## Supporting information


Supplementary Material 1.

Supplementary Table S1.

Supplementary Table S2.

Supplementary Table S3.

## Data Availability

Not applicable.

## References

[1] Iwama T , Tamura K , Morita T , et al. A clinical overview of familial adenomatous polyposis derived from the database of the polyposis registry of Japan. Int J Clin Oncol. 2004;9(4):308‐316. 15375708 10.1007/s10147-004-0414-4

[2] Murata M , Utsunomiya J , Iwama T , Tanimura M . Frequency of adenomatosis‐coli in Japan. Jpn J Hum Genet. 1981;26(1):19‐30. 10.1007/BF01871370 7265541

[3] Vasen HF , Moslein G , Alonso A , et al. Guidelines for the clinical management of familial adenomatous polyposis (FAP). Gut. 2008;57(5):704‐713. 18194984 10.1136/gut.2007.136127

[4] Hill MJ , Morson BC , Bussey HJ . Aetiology of adenoma—carcinoma sequence in large bowel. Lancet. 1978;1(8058):245‐247. 74668 10.1016/s0140-6736(78)90487-7

[5] Tomita N , Ishida H , Tanakaya K , et al. Japanese Society for Cancer of the Colon and Rectum (JSCCR) guidelines 2020 for the clinical practice of hereditary colorectal cancer. Int J Clin Oncol. 2021;26(8):1353‐1419. 34185173 10.1007/s10147-021-01881-4 PMC8286959

[6] Aziz O , Athanasiou T , Fazio VW , et al. Meta‐analysis of observational studies of ileorectal versus ileal pouch‐anal anastomosis for familial adenomatous polyposis. Br J Surg. 2006;93(4):407‐417. 16511903 10.1002/bjs.5276

[7] Nieuwenhuis MH , Douma KF , Bleiker EM , Bemelman WA , Aaronson NK , Vasen HF . Female fertility after colorectal surgery for familial adenomatous polyposis: a nationwide cross‐sectional study. Ann Surg. 2010;252(2):341‐344. 20622653 10.1097/SLA.0b013e3181e9829f

[8] Saito Y , Hinoi T , Ueno H , et al. Risk factors for the development of desmoid tumor after colectomy in patients with familial adenomatous polyposis: multicenter retrospective cohort study in Japan. Ann Surg Oncol. 2016;23(Suppl 4):559‐565. 27387679 10.1245/s10434-016-5380-3

[9] Konishi T , Ishida H , Ueno H , et al. Feasibility of laparoscopic total proctocolectomy with ileal pouch‐anal anastomosis and total colectomy with ileorectal anastomosis for familial adenomatous polyposis: results of a nationwide multicenter study. Int J Clin Oncol. 2016;21(5):953‐961. 27095110 10.1007/s10147-016-0977-x

[10] Ishikawa H , Mutoh M , Iwama T , et al. Endoscopic management of familial adenomatous polyposis in patients refusing colectomy. Endoscopy. 2016;48(1):51‐55. 26352809 10.1055/s-0034-1392774

[11] Ishikawa H , Yamada M , Sato Y , et al. Intensive endoscopic resection for downstaging of polyp burden in patients with familial adenomatous polyposis (J‐FAPP study III): a multicenter prospective interventional study. Endoscopy. 2023;55(4):344‐352. doi:10.1055/a-1945-9120 36216266 PMC10060053

[12] Cole BF , Logan RF , Halabi S , et al. Aspirin for the chemoprevention of colorectal adenomas: meta‐analysis of the randomized trials. J Natl Cancer Inst. 2009;101(4):256‐266. 19211452 10.1093/jnci/djn485 PMC5975663

[13] Ishikawa H , Mutoh M , Suzuki S , et al. The preventive effects of low‐dose enteric‐coated aspirin tablets on the development of colorectal tumours in Asian patients: a randomised trial. Gut. 2014;63(11):1755‐1759. 24488498 10.1136/gutjnl-2013-305827

[14] Ishikawa H , Mutoh M , Sato Y , et al. Chemoprevention with low‐dose aspirin, mesalazine, or both in patients with familial adenomatous polyposis without previous colectomy (J‐FAPP study IV): a multicentre, double‐blind, randomised, two‐by‐two factorial design trial. Lancet Gastroenterol Hepatol. 2021;6(6):474‐481. 33812492 10.1016/S2468-1253(21)00018-2

[15] DuPont AW , Arguedas MR , Wilcox CM . Aspirin chemoprevention in patients with increased risk for colorectal cancer: a cost‐effectiveness analysis. Aliment Pharmacol Ther. 2007;26(3):431‐441. 17635378 10.1111/j.1365-2036.2007.03380.x

[16] Cooper K , Squires H , Carroll C , et al. Chemoprevention of colorectal cancer: systematic review and economic evaluation. Health Technol Assess. 2010;14(32):1‐206. 10.3310/hta14320 20594533

[17] Owens DK , Whitlock EP , Henderson J , et al. Use of decision models in the development of evidence‐based clinical preventive services recommendations: methods of the U.S. preventive services task force. Ann Intern Med. 2016;165(7):501‐508. 27379742 10.7326/M15-2531

[18] Statistics BureauMinistry of Internal Affairs and Communications of Japan . Population Estimates of Japan 1920–2000. Available from: https://www.e‐stat.go.jp/en/stat‐search/files?page=1&layout=datalist&toukei=00200524&tstat=000000090001&cycle=0&tclass1=000000090004&tclass2=000000090005&tclass3val=0

[19] National Institute of Population and Social Security Research . The Japanese Mortality Database. Available from: http://www.ipss.go.jp/p‐toukei/JMD/00/index‐en.html

[20] National Cancer Center . Cancer Information Services. Available from: https://ganjoho.jp/reg_stat/statistics/dl/index.html

[21] Lansdorp‐Vogelaar I , Kuntz KM , Knudsen AB , Wilschut JA , Zauber AG , van Ballegooijen M . Stool DNA testing to screen for colorectal cancer in the Medicare population: a cost‐effectiveness analysis. Ann Intern Med. 2010;153(6):368‐377. 20855801 10.1059/0003-4819-153-6-201009210-00004 PMC3578600

[22] Rutter CM , Savarino JE . An evidence‐based microsimulation model for colorectal cancer: validation and application. Cancer Epidemiol Biomarkers Prev. 2010;19(8):1992‐2002. 20647403 10.1158/1055-9965.EPI-09-0954 PMC2919657

[23] Ito Y , Miyashiro I , Ito H , et al. Long‐term survival and conditional survival of cancer patients in Japan using population‐based cancer registry data. Cancer Sci. 2014;105(11):1480‐1486. 25183551 10.1111/cas.12525 PMC4462379

[24] Bertario L , Russo A , Sala P , et al. Survival of patients with hereditary colorectal cancer: comparison of HNPCC and colorectal cancer in FAP patients with sporadic colorectal cancer. Int J Cancer. 1999;80(2):183‐187. 9935197 10.1002/(sici)1097-0215(19990118)80:2<183::aid-ijc4>3.0.co;2-w

[25] Sweet A , Lee D , Gairy K , Phiri D , Reason T , Lock K . The impact of CT colonography for colorectal cancer screening on the UK NHS: costs, healthcare resources and health outcomes. Appl Health Econ Health Policy. 2011;9(1):51‐64. 21174482 10.2165/11588110-000000000-00000

[26] Iwama T , Mishima Y , Utsunomiya J . The impact of familial adenomatous polyposis on the tumorigenesis and mortality at the several organs. Its rational treatment. Ann Surg. 1993;217(2):101‐108. 8382467 10.1097/00000658-199302000-00002 PMC1242747

[27] Briggs A , Sculpher M , Claxton K . Decision Modelling for Health Economic Evaluation. Oxford University Press; 2006.

[28] World Health O , Baltussen RMPM , Adam T , et al. Making Choices in Health: WHO Guide to Cost‐Effectiveness Analysis/Edited by T. Tan‐Torres Edejer … [et al]. World Health Organization; 2003.

[29] Igarashi A , Goto R , Yoneyama‐Hirozane M . Willingness to pay for QALY: perspectives and contexts in Japan. J Med Econ. 2019;22(10):1041‐1046. 31262236 10.1080/13696998.2019.1639186

[30] Husereau D , Drummond M , Petrou S , et al. Consolidated Health economic evaluation reporting standards (CHEERS) statement. Value Health. 2013;16(2):e1‐e5. 23538200 10.1016/j.jval.2013.02.010

[31] Lloyd KE , Hall LH , King N , et al. Aspirin use for cancer prevention: a systematic review of public, patient and healthcare provider attitudes and adherence behaviours. Prev Med. 2022;154:106872. 34762964 10.1016/j.ypmed.2021.106872 PMC8803547

[32] Ishikawa H , Wakabayashi K , Suzuki S , et al. Preventive effects of low‐dose aspirin on colorectal adenoma growth in patients with familial adenomatous polyposis: double‐blind, randomized clinical trial. Cancer Med. 2013;2(1):50‐56. 24133627 10.1002/cam4.46 PMC3797560

[33] Burn J , Bishop DT , Chapman PD , et al. A randomized placebo‐controlled prevention trial of aspirin and/or resistant starch in young people with familial adenomatous polyposis. Cancer Prev Res (Phila). 2011;4(5):655‐665. 21543343 10.1158/1940-6207.CAPR-11-0106 PMC3092423

[34] Li H , Zhu F , Boardman LA , et al. Aspirin prevents colorectal cancer by normalizing EGFR expression. EBioMedicine. 2015;2(5):447‐455. 26097892 10.1016/j.ebiom.2015.03.019 PMC4469241

[35] Ishikawa H , J‐FAPP Study III Group . Introduction of a multi‐centered clinical trial ‐ intensive downstaging polypectomy ro prevent colorectal cancer in FAP patients [in Japanese]. 家族性腫瘍 [Kazokusei Shuyou]. 2014;14(2):26‐28.

[36] Ness RM , Holmes AM , Klein R , Dittus R . Utility valuations for outcome states of colorectal cancer. Am J Gastroenterol. 1999;94(6):1650‐1657. 10364039 10.1111/j.1572-0241.1999.01157.x

[37] Dossa F , Morris AM , Wilson AR , Baxter NN . Life after surgery: surgeon assessments of quality of life among patients with familial adenomatous polyposis. Dis Colon Rectum. 2018;61(10):1217‐1222. 30192330 10.1097/DCR.0000000000001146

[38] Takeuchi Y , Hamada K , Nakahira H , et al. Efficacy and safety of intensive downstaging polypectomy (IDP) for multiple duodenal adenomas in patients with familial adenomatous polyposis: a prospective cohort study. Endoscopy. 2023;55(6):515‐523. 36410678 10.1055/a-1983-5963

[39] Bibbins‐Domingo K . Aspirin use for the primary prevention of cardiovascular disease and colorectal cancer: U.S. preventive services task force recommendation statement. Ann Intern Med. 2016;164(12):836‐845. 27064677 10.7326/M16-0577

[40] Davidson KW , Barry MJ , Mangione CM , et al. Aspirin use to prevent cardiovascular disease: US preventive services task force recommendation statement. JAMA. 2022;327(16):1577‐1584. 35471505 10.1001/jama.2022.4983

[41] Hashimoto H , Ikegami N , Shibuya K , et al. Cost containment and quality of care in Japan: is there a trade‐off? Lancet. 2011;378(9797):1174‐1182. 21885098 10.1016/S0140-6736(11)60987-2

[42] Xie M , Chen Y , Wei W , et al. Does ileoanal pouch surgery increase the risk of desmoid in patients with familial adenomatous polyposis? Int J Colorectal Dis. 2020;35(8):1599‐1605. 32435838 10.1007/s00384-020-03578-y

[43] Watanabe Y , Ishida H , Baba H , et al. Pancreas‐sparing total duodenectomy for Spigelman stage IV duodenal polyposis associated with familial adenomatous polyposis: experience of 10 cases at a single institution. Fam Cancer. 2017;16(1):91‐98. 27655252 10.1007/s10689-016-9932-2

[44] Augustin T , Moslim MA , Tang A , Walsh RM . Tailored surgical treatment of duodenal polyposis in familial adenomatous polyposis syndrome. Surgery. 2018;163(3):594‐599. 29331402 10.1016/j.surg.2017.10.035

[45] Sekiguchi M , Igarashi A , Matsuda T , et al. Optimal use of colonoscopy and fecal immunochemical test for population‐based colorectal cancer screening: a cost‐effectiveness analysis using Japanese data. Jpn J Clin Oncol. 2016;46(2):116‐125. 26685321 10.1093/jjco/hyv186

[46] Yoshino S , Igarashi Y , OHara H , et al. Report on the 5th survey on complications associated with endoscopy—2003 to 2007—[in Japanese]. 日本消化器内視鏡学会雑誌 [Nihon Shokaki Naishikyo Gakkai Zasshi]. 2010;52(1):95‐103.

[47] Tajika M , Tanaka T , Ishihara M , et al. Long‐term outcomes of metachronous neoplasms in the ileal pouch and rectum after surgical treatment in patients with familial adenomatous polyposis. Endosc Int Open. 2019;7(5):E691‐E698. 31073536 10.1055/a-0849-9465 PMC6506341

[48] National Collaborating Centre for Chronic C . National Institute for Health and Clinical Excellence: Guidance. Osteoarthritis: National Clinical Guideline for Care and Management in Adults. Royal College of Physicians (UK) Copyright © 2008, Royal College of Physicians of London; 2008.

[49] Sekiguchi M , Igarashi A , Sakamoto T , Saito Y , Esaki M , Matsuda T . Cost‐effectiveness analysis of colorectal cancer screening using colonoscopy, fecal immunochemical test, and risk score. J Gastroenterol Hepatol. 2020;35(9):1555‐1561. 32167186 10.1111/jgh.15033

