# Supplementary material for: Cost‐effectiveness of preventive aspirin use and intensive downstaging polypectomy in patients with familial adenomatous polyposis: A microsimulation modeling study
Source: Cancer Med. 2023 Aug 30;12(18):19137–48. doi: 10.1002/cam4.6488 (PMC10557886; doi:10.1002/cam4.6488)
Supplement: Supplementary file 1 — Supplementary Material 1. [file CAM4-12-19137-s003.docx]

**Supplemental Document 1. Description of the microsimulation model**

This microsimulation model uses the Markov Chain Monte Carlo method to simulate all events in the program. The model consists of three components:

1. Demographic component

2. Natural history component, and

3. Intervention component.

These components are not physically separate in the model, but it is helpful to understand the structure of the model when presented this way.

1. Demographic component

The demographic component consists of the cohort of the Japanese population by sex and age ranging between 0 and 85+ years who were alive in 1963 (1) and lasts for 40 years (annual cycles). The 1963 cohort will then experience the background mortality by age and sex of the corresponding year as they age. Background mortality from all causes was obtained from the Japanese Mortality Database 1920-2016 (2), and colorectal cancer (CRC) mortality of the corresponding year by age and sex was obtained from Cancer Information Services (3). We assumed that death from colorectal cancer and death from other causes are independent of each other. Background mortality rates and colorectal cancer mortality rates were converted to probabilities of dying in a given year by age and sex using the following formula:

p=1-exp(-rt)

where:

p=probability;

r= the instantaneous rate provided that it is constant over the period of interest (t) (4). By subtracting the probability of dying due to colorectal cancer from the probability of dying from all causes, we estimated the probability of dying from causes other than colorectal cancer for a given year by age and sex.

2. Natural History Component

The Natural History Component is a part of the microsimulation model, which simulates colorectal cancer histories (natural histories) for each individual separately. We based our natural history model on the adenoma-carcinoma sequence (5), with the modification that all FAP patients enter into the model with one or more high-risk adenomas at the time of diagnosis (6). Some of the adenomas develop into colorectal cancer according to the age-specific incidence of CRC depending on the natural history parameters. The survivorship of a person once an adenoma has developed into clinical colorectal cancer depends on sex, cancer stage at diagnosis and years after diagnosis (7).

3. Intervention Component

The intervention component is simultaneously run with the Natural History Component, making detection of adenomas and carcinomas in different states possible. Interventions in the model potentially affect all preclinical disease stages, resulting either in removal of an adenoma, or in prevention of CRC or early detection of a preclinical carcinoma, possibly in an earlier stage, resulting in a favorable stage shift and thus improved prognosis. The effectiveness and harms of an intervention depend on the intervention parameters.

**Reference**

1. Statistics Bureau, Ministry of Internal Affairs and Communications of Japan. Population Estimates of Japan 1920 - 2000 [Available from: https://www.e-stat.go.jp/en/stat-search/files?page=1&layout=datalist&toukei=00200524&tstat=000000090001&cycle=0&tclass1=000000090004&tclass2=000000090005&tclass3val=0.

2. National Institute of Population and Social Security Research. The Japanese Mortality Database [Available from: http://www.ipss.go.jp/p-toukei/JMD/00/index-en.html.

3. National Cancer Center. Cancer Information Services [Available from: https://ganjoho.jp/reg_stat/statistics/dl/index.html.

4. Briggs A, Sculpher M, Claxton K. Decision modelling for health economic evaluation: Oup Oxford; 2006.

5. Hill MJ, Morson BC, Bussey HJ. Aetiology of adenoma--carcinoma sequence in large bowel. Lancet. 1978;1(8058):245-7.

6. Iwama T, Tamura K, Morita T, Hirai T, Hasegawa H, Koizumi K, et al. A clinical overview of familial adenomatous polyposis derived from the database of the Polyposis Registry of Japan. Int J Clin Oncol. 2004;9(4):308-16.

7. Ito Y, Miyashiro I, Ito H, Hosono S, Chihara D, Nakata-Yamada K, et al. Long-term survival and conditional survival of cancer patients in Japan using population-based cancer registry data. Cancer Sci. 2014;105(11):1480-6.
