# Supplementary material for: Cost‐effectiveness of preventive aspirin use and intensive downstaging polypectomy in patients with familial adenomatous polyposis: A microsimulation modeling study
Source: Cancer Med. 2023 Aug 30;12(18):19137–48. doi: 10.1002/cam4.6488 (PMC10557886; doi:10.1002/cam4.6488)
Supplement: Supplementary file 2 — Supplementary Table S1. [file CAM4-12-19137-s002.docx]

**Supplemental Table 1. Calibrated and original data for the natural history model**

| **Input parameter** | **Calibrated^a^** | **Original** | **Source** |
| --- | --- | --- | --- |
| From high-risk polyp to preclinical stage I CRC in FAP patients (<30 years old) | 0.01598 | 0.21964 | (1) |
| From high-risk polyp to preclinical stage I CRC in FAP patients (30+ years old) | 0.11376 | 0.21964 | (1) |
| From preclinical stage I to preclinical stage II CRC | 0.24022 | 0.21022 | (1) |
| From preclinical stage II to preclinical stage III CRC | 0.22290 | 0.19023 | (1) |
| From preclinical stage III to preclinical stage IV CRC | 0.27487 | 0.15465 | (1) |
| From preclinical stage I to clinical stage I CRC | 0.07202 | 0.06500 | (2) |
| From preclinical stage II to clinical stage II CRC | 0.11011 | 0.26000 | (2) |
| From preclinical stage III to clinical stage III CRC | 0.18989 | 0.46000 | (2) |
| From preclinical stage IV to clinical stage IV CRC | 0.87577 | 0.92000 | (2) |

a Calibrated parameter values were used in the final model.

**Reference**

1. Bertario L, Russo A, Sala P, Eboli M, Radice P, Presciuttini S, et al. Survival of patients with hereditary colorectal cancer: comparison of HNPCC and colorectal cancer in FAP patients with sporadic colorectal cancer. Int J Cancer. 1999;80(2):183-7.

2. Sweet A, Lee D, Gairy K, Phiri D, Reason T, Lock K. The impact of CT colonography for colorectal cancer screening on the UK NHS: costs, healthcare resources and health outcomes. Appl Health Econ Health Policy. 2011;9(1):51-64.
