# Supplementary material for: Cost‐effectiveness of preventive aspirin use and intensive downstaging polypectomy in patients with familial adenomatous polyposis: A microsimulation modeling study
Source: Cancer Med. 2023 Aug 30;12(18):19137–48. doi: 10.1002/cam4.6488 (PMC10557886; doi:10.1002/cam4.6488)
Supplement: Supplementary file 4 — Supplementary Table S3. [file CAM4-12-19137-s004.docx]

**Supplemental Table 3. Sensitivity analysis of incremental cost-effectiveness of colorectal cancer prevention strategies in FAP patients with varying rates of aspirin adherence**

|  | 80% | | |  | 85% | | |  | 90% | | |  | 95% | | |
| --- | --- | --- | --- | --- | --- | --- | --- | --- | --- | --- | --- | --- | --- | --- | --- |
|  | QALYs  per person^a^ | Average cost per person^a^ | ICER per QALY gained |  | QALYs  per person^a^ | Average cost per person^a^ | ICER per QALY gained |  | QALYs  per person^a^ | Average cost per person^a^ | ICER per QALY gained |  | QALYs  per person^a^ | Average cost per person^a^ | ICER per QALY gained |
| No intervention | 19.19 | 10,972 | - |  | 19.19 | 10,972 | - |  | 19.19 | 10,972 | - |  | 19.19 | 10,972 | - |
| Strategy 1 (Polypectomy) | 21.01 | 19,314 | Dominated^b^ |  | 21.01 | 19,314 | Dominated^b^ |  | 21.01 | 19,314 | Dominated^b^ |  | 21.01 | 19,314 | Dominated^b^ |
| Strategy 2 (Polypectomy & Aspirin) | 21.31 | 12,949 | 932^c^ |  | 21.33 | 12,523 | 725^c^ |  | 21.34 | 12,062 | 508^c^ |  | 21.37 | 11,620 | 297^c^ |
| Strategy 3 (IPAA) | 21.43 | 19,096 | 52,180^d^ |  | 21.43 | 19,096 | 65,516^d^ |  | 21.43 | 19,096 | 76,472^d^ |  | 21.43 | 19,096 | 121,418^d^ |

QALY, Quality-Adjusted Life-Year; CRC, colorectal cancer; ICER, Incremental Cost-Effectiveness Ratio; IPAA, Ileal Pouch-Anal Anastomosis

a Discounted at an annual rate of 3%.

b Dominated with a greater cost and fewer QALYs than Strategy 2.

c Compared against no intervention.

d Compared against Strategy 2.
